# Delayed skeletal maturity in dwarf, medium and giant Pleistocene insular deer (*Candiacervus*) indicating a slower life history regardless size shift

**DOI:** 10.1186/s13358-025-00413-1

**Published:** 2025-12-23

**Authors:** Felix Snoodijk, Teresa Calderón, George A. Lyras, Alexandra A. E. van der Geer

**Affiliations:** 1 Institute of Biology, Leiden University, PO Box 9505, Sylviusweg 72, 2333 BE Leiden, the Netherlands; 2 Naturalis Biodiversity Center, PO Box 9517, 2300 RA Leiden, the Netherlands; 3 Departamento de Ciencias de La Tierra, and Instituto Universitario de Investigación en Ciencias Ambientales de Aragón (IUCA) , Universidad de Zaragoza, 50009 Zaragoza, Spain; 4 National and Kapodistrian University of Athens, Panepistimiopolois, 15784 Zografos, Athens, Greece

**Keywords:** Crete, *Dama dama*, Dwarfism, Gigantism, Bone histology

## Abstract

Insular conditions, such as reduced levels of resources, predation and competition, may affect life history traits of island mammal species, promoting a shift towards a slow life history. This pattern has been found in several species of insular dwarf deer but has not been tested for island deer that were subject to gigantism, which may evolve opposite life history shifts. The Pleistocene deer genus of Crete (Greece), *Candiacervus,* provides an ideal case to test this potential difference, because this endemic genus comprised dwarf as well as giant forms in an otherwise depauperate mammalian fauna. Here, we tested maturation patterns in this genus using a palaeohistological approach. We found that small, medium and larger forms of *Candiacervus* all demonstrated delayed skeletal maturity compared to their putative ancestor fallow deer (*Dama dama*) as well as similar-sized mainland deer. This is the first report showing that insular deer that are either subject to gigantism or retained ancestral size, also displayed adaptations to a slow life history similar to insular deer that underwent dwarfism. This research contributes to our understanding of how insular conditions may influence life history patterns in a species radiation of deer.

## Introduction

During the late Middle and Late Pleistocene, the island of Crete (Greece) was home to an impoverished mammalian fauna including a shrew, a giant mouse, an otter, several deer and a dwarf elephant (Lyras et al., 2022; van der Geer et al., 2021). The deer all belong to the extinct genus *Candiacervus* Kuss, 1975 endemic to the island, which contains eight species distributed over six different size classes (de Vos, 1979; van der Geer, 2018). The body mass and shoulder height between *Candiacervus* species vary between 27.8 kg and 40 cm for the smallest size (*C. ropalophorus* de Vos, 1984; referred to as size 1 in de Vos, 1984) and 245.4 kg and 165 cm for the largest size (*C. major* Capasso Barbato & Petronio, 1986; referred to as size 6 in de Vos, 1984) (Besiou et al., 2022; Lyras et al., 2022). Based on anatomical differences, the *Candiacervus* taxa likely occupied different niches and thus experienced limited interspecific competition (de Vos & van der Geer, 2002). Furthermore, *Candiacervus* had no terrestrial predators (van der Geer et al., 2014), and potential ecological competition with species outside of the genus was limited to the dwarf elephant (Palombo & Zedda, 2016).

Island mammals, like *Candiacervus*, often show morphological and physiological adaptations in response to island conditions, including resource limitation, reduced ecologically relevant predation and low interspecific competition (Köhler, 2010; Lomolino et al., 2012, 2013; Palkovacs, 2003). In general, larger insular mammal species gradually evolve into smaller forms (dwarfism), while smaller insular mammal species gradually evolve into larger forms (gigantism), as predicted by the ecogeographic pattern often termed the ‘island rule’ (Foster, 1965; Lomolino, 1985, 2005; van Valen, 1973). The smallest size and the three species of the following size class (*C. devosi*, *C. listeri* & *C. reumeri* van der Geer, 2018; together referred to as size 2 in de Vos, 1984) are considered dwarf taxa in the sense of the island rule (van der Geer et al., 2014). The medium-sized taxa *C. cretensis* (Simonelli, 1907) and *C. rethymnensis* Kuss, 1975 (referred to as sizes 3 and 4, respectively, in de Vos, 1984) are comparable in size to the putative ancestral *Dama* (Besiou et al., 2022; van der Geer, 2018). The largest taxa are represented by *C. dorothensis* (Capasso Barbato, 1992; referred to as size 5 in de Vos, 1984) and *C. major*, which were subject to gigantism (Besiou et al., 2022; Palombo & Zedda, 2022).

Island conditions may also affect life history traits of mammals, like growth rate, age of maturity and longevity (Hayashi et al., 2023; Köhler & Moyà-Solà, 2009; Köhler et al., 2021; Long et al., 2019; Palkovacs, 2003). A slow life history seems to be a general trend among dwarfed island herbivores (Hayashi et al., 2023; Köhler & Moyà-Solà, 2009; Köhler et al., 2021; Long et al., 2019; Palkovacs, 2003). Somewhat counterintuitively, a similar shift in life history towards a slower pace of life has also been attested in giant forms of small mammals (Fernández-Bejarano et al., 2024; Hayashi et al., 2025; Moncunill-Solé et al., 2016; Orlandi-Oliveras et al., 2016). Up to now, studies on the dwarf *Candiacervus* indicate slow life history traits like low growth rates, late skeletal maturity and extended longevity as a response to insular conditions (Kolb et al., 2015; Lyras et al., 2016, 2019; Miszkiewicz & van der Geer, 2022; Palombo & Zedda, 2016; van der Geer et al., 2006, 2014). Skeletal maturity differs from sexual maturity and marks the moment when the epiphyses are completely fused and growth slows down and ceases, as inferred from red deer femora (Calderón et al., 2019). Information on the larger *Candiacervus* is, however, lacking, apart from an empirical study on one, potentially pathological, individual of the giant size six, where an anomalous length growth was recorded in combination with minimal width increase in two long bones, resulting in an extreme thinning of the diaphyseal cortical bone (Palombo & Zedda, 2022).

Changes in life history are usually recorded in bone growth (Bromage et al., 2009; Castanet, 2006; Hayashi et al., 2023; Köhler & Moyà-Solà, 2009; Köhler et al., 2021; Marín-Moratalla et al., 2011). Therefore, the analysis of bone microstructure allows reconstruction of evolutionary shifts in these life history traits of fossil mammals (e.g., Amson et al., 2015; Hayashi et al., 2023; Köhler & Moyà-Solà, 2009; Köhler et al., 2021; Kolb et al., 2015; Marín-Moratalla et al., 2011; Moncunill-Solé et al., 2016; Nacarino-Meneses & Orlandi-Oliveras, 2021; Orlandi-Oliveras et al., 2016, 2018). Shifts in life history traits may differ in degree and direction between dwarf and giant forms, following patterns seen in mainland taxa, where small species tend to have a fast life history, and large species a slow life history, known as the fast–slow continuum of life-history variation (Promislow & Harvey, 1990). The genus *Candiacervus* provides a unique case to test for this, because this genus contains both dwarf and giant forms that lived in sympatry under presumably the same geographic and climatic conditions (van der Geer et al., 2021), limiting the confounding effect of differences in these variables. In addition to this, histological studies on the life history traits of large-sized herbivorous insular mammals, which are subject to gigantism, are scarce (e.g., Palombo & Zedda, 2022).

In this study, the life history of the dwarf, medium and giant taxa of *Candiacervus* is examined. We hypothesize that all *Candiacervus* sizes evolved towards a slower life history because they all shared a predator-free habitat with limited resources and limited interspecific competition. To test this, we analysed maturation patterns preserved in long bones using palaeohistology. This research will increase our understanding of the evolution of sympatric and congeneric differently sized forms under ecological release from predation and probably increased interspecific competition on large islands.

## Materials and methods

**Fig. 1:**
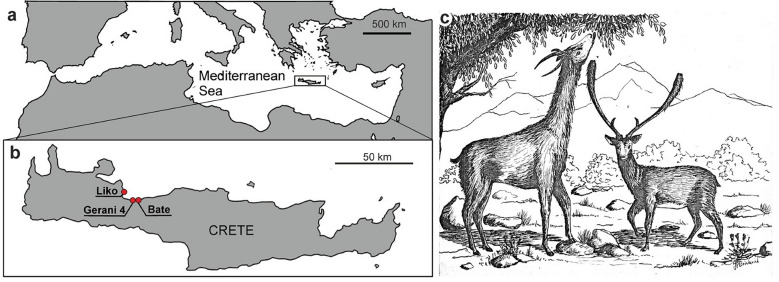
Localities of sampled *Candiacervus* specimens and a reconstruction of two Cretan deer taxa. **a** Map of the Mediterranean and **b** close-up of Crete with red dots indicating different caves (Modified after van der Geer, 2018). **c** Reconstructions of giant *C. dorothensis* (left) and dwarf *C. ropalophorus* (right). Drawing by Hans Brinkerink.

Five femora of *Candiacervus* size one (*C. ropalophorus*), two of size two (*C. listeri, C. reumeri, C. devosi*), one of size three (*C. cretensis*) and two of size five (*C. dorothensis*), and two femora of adult fallow deer (*Dama dama* Linnaeus, 1758)*,* were sampled (Table 1). All specimens preserved at least one fused epiphysis, proving skeletal maturity (following Calderón et al., 2019). We here choose fallow deer (*D. dama*) as a comparative species, because this taxon is phylogenetically the closest living relative of *Candiacervus* (van der Geer, 2018). The three species referred to as size two cannot be distinguished based on postcranial material or locality (van der Geer, 2018). The *Candiacervus* specimens are all of Late Pleistocene age and were obtained from the caves Liko, Gerani 4 and Bate on Crete (Fig. 1). Recent *D. dama* bones were collected from Rhodos (Greece) and Wildnispark Zürich (Switzerland) (Table 1).

**Fig. 2:**
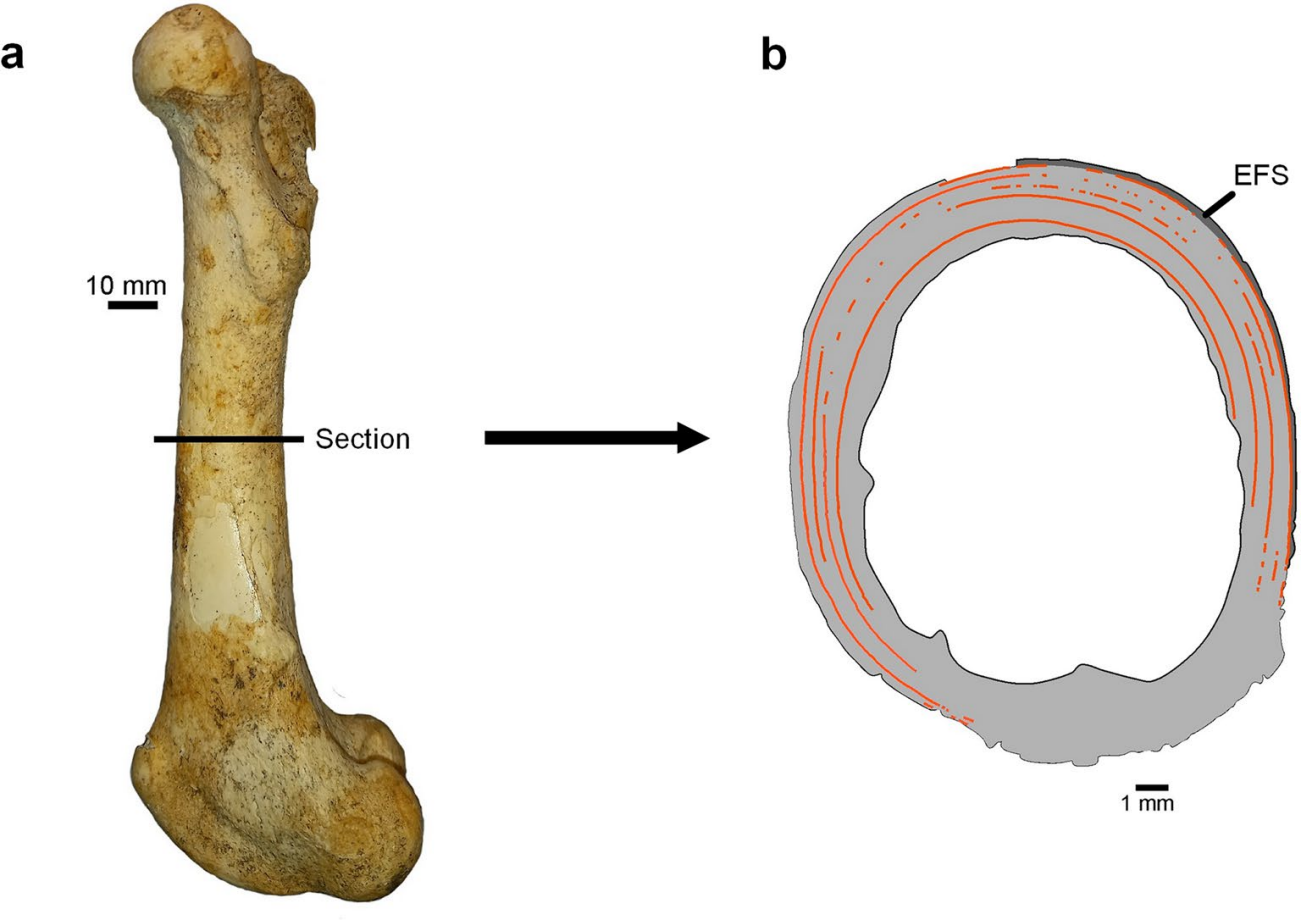
*Candiacervus ropalophorus* femur with schematic representation of its cross-section. **a** Lateral view of specimen RGM 439541 with the sectioning site indicated. **b** Schematic representation of section 2255 from the same specimen with orange lines representing lines of arrested growth (LAGs) and the dark grey colour indicating the external fundamental system (EFS)

Thin sections of the femora of nine specimens (8 from *Candiacervus*, 1 from *D. dama*; Table 1) were prepared following the methods in Cuccu et al. (2024). In summary, the process consisted of extracting approximately two centimetres of the middle part of the diaphysis with a Dremel (Fig. 2a), after which the extant specimens were stepwise dehydrated. All segments were then embedded in epoxy resin (Araldite 2020) and cut transversely with a low-speed diamond saw (Struers Accutom 2). Subsequently, the samples were attached to microscopic slides, after which they were cut again to create cross-sections with a thickness of approximately 1700 µm. This thickness was step-by-step reduced to 100 µm with gradually finer polishing papers, while regularly checking the visibility of the tissue. Three specimens had already been prepared earlier and used in Kolb et al. (2015) (Table 1). In this study, these three thin sections were re-analysed. All the slides were studied with a Leica DMRP microscope (2.5x, 5x, 10x) under transmitted and cross-polarized light to visualize the bone tissue.

In most tetrapods, two different types of cortical bone can be distinguished: primary (lamellar bone, parallel-fibred bone and fibrolamellar complex/woven-fibered bone) and secondary bone (endosteal lamellar bone and Haversian bone) (Francillon-Vieillot et al., 1990; Huttenlocker et al., 2013). Disorganized tissue like fibrolamellar complex (FLC) gets deposited quickly, while organized lamellar/parallel-fibred bone is deposited at a slow rate (Currey, 2002; de Margerie et al., 2002; Francillon-Vieillot et al., 1990). In most mammals, several bird species and large dinosaurs, the dominant tissue type is fibrolamellar bone (Castanet et al., 2000; Currey, 2002; Horner et al., 2005). Fibrolamellar bone can display multiple orientations of vascularization like radial, reticular, plexiform, laminar and longitudinal, where radial indicates the fastest growth and longitudinal the slowest (Francillon-Vieillot et al., 1990; Huttenlocker et al., 2013). Sometimes, a band of parallel-fibred avascular tissue can be distinguished in the outer cortex (sub-periosteal area), referred to as the external fundamental system (EFS) (Huttenlocker et al., 2013). In deer, the transition of the fast-growing FLC to the slow-growing EFS in the femur denotes the attainment of skeletal maturity, as indicated by Calderón et al. (2019) in red deer. The bone tissue of the FLC and EFS is often interrupted by growth marks (Huttenlocker et al., 2013). A growth mark represents a pause or decrease in bone deposition and can reflect cyclical or single events (Huttenlocker et al., 2013; Woodward et al., 2013). A growth mark representing complete growth cessation is here referred to as a line of arrested growth (LAG) (following Huttenlocker et al., 2013; Woodward et al., 2013). We assume that the deposition of LAGs, which can be traced around the entire circumference of the cortex, is cyclical and represents annual arrest of bone growth (following Köhler et al., 2012; Woodward et al., 2013). These cyclical lines have been used for estimating age. However, caution is required, as in mature individuals these lines can be disturbed by medullary expansion, resorption and remodelling of the bone tissue or are closely packed in the EFS (Kolb et al., 2015; Woodward et al., 2013). As confirmed in red deer, the total number of LAGs in the femur does not reflect the exact age of an adult individual (Calderón et al., 2019). Instead, the number of LAGs only corresponds to the age before deposition of the EFS (Calderón et al., 2019), which makes the count of LAGs within the FLC, including the LAG separating the FLC from the EFS, an accurate proxy for calculating the age of attainment of skeletal maturity in deer. The first LAG is located at the innermost part of the cortex. In this study, the majority of the LAGs within the FLC were counted on the anterior side of the cortex, where they had not been erased due to medullary expansion (Fig. 2b).

## Results

**Fig. 3:**
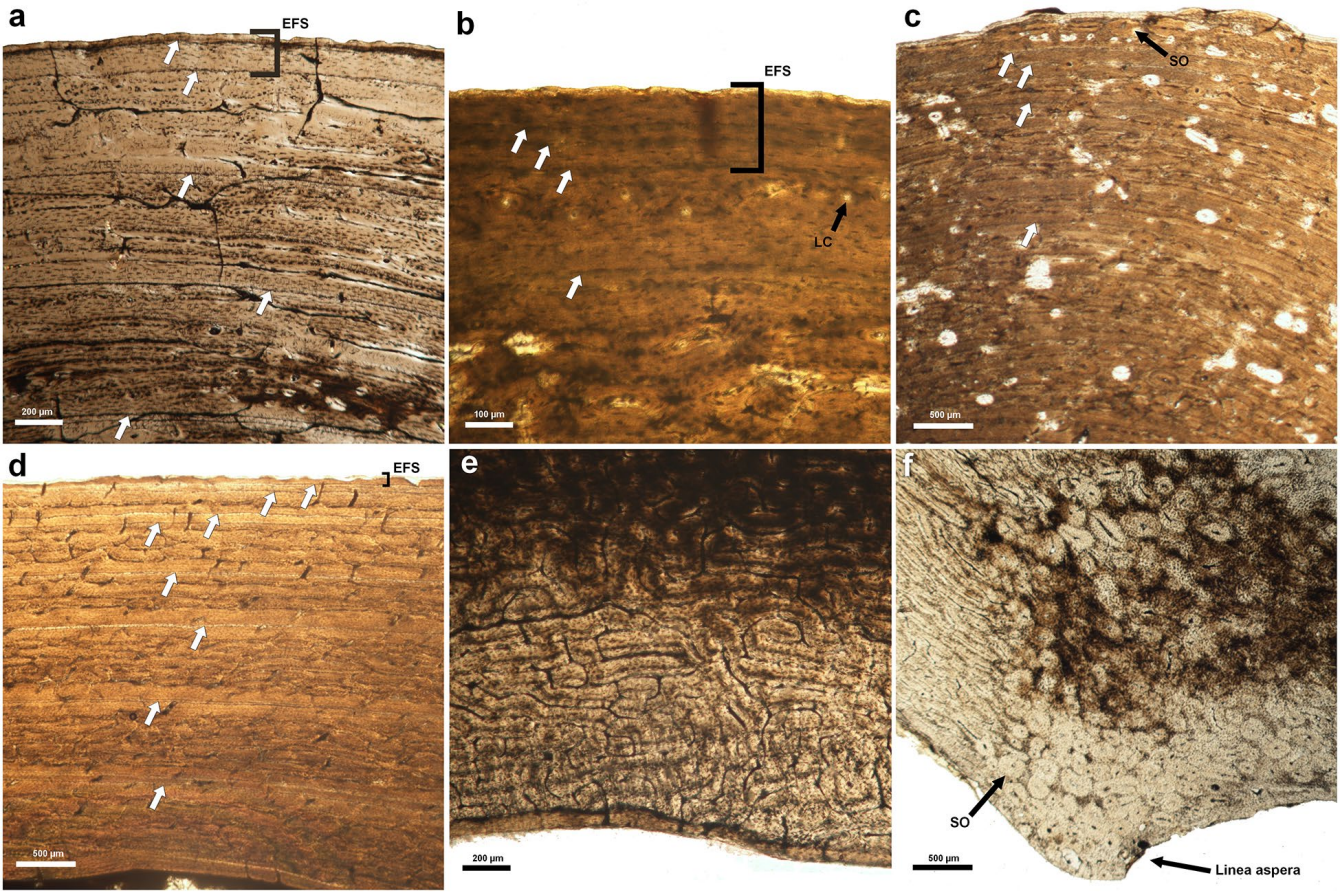
Bone tissue of *Candiacervus* and *Dama dama*. **a** Bone tissue in the anterior part of the cortex of *Candiacervus* size one (2294) exhibits predominantly laminar tissue and some scattered longitudinal canals. The first to fourth lines of arrested growth (LAGs), with the first LAG formed most internally, are visible and surrounded by a more organized tissue within the fibrolamellar complex (FLC). In the external fundamental system (EFS) one distinguishable LAG is visible. **b** Bone tissue in the anterior part of the cortex of *Candiacervus* size two (4805) exhibiting the fifth and sixth LAGs within the FLC and a pronounced EFS showing two extra LAGs. Several longitudinal canals (LC) are present between the fifth and sixth LAGs. **c** Bone tissue in the anterior part of the cortex of *Candiacervus* size three (3701) showing the second to fifth LAGs surrounded by slow-growing tissue within the FLC with predominantly laminar and plexiform vascularisation. Scattered secondary osteons (SO) obscure the growth record even within the sub-periosteal area. **d** Bone tissue in the lateral part of the cortex of *Candiacervus* size five (2015) exhibiting the second to ninth LAGs surrounded by slow-growing tissue within the FLC. The FLC displays primarily plexiform and laminar vascularisation. (a-d) The white arrows indicate the position of the LAGs. **e** Bone tissue in the medial part of the inner cortex of *D. dama* (PHZ 554) exhibiting mainly reticular tissue. **f** Linea aspera of the cortex of *D. dama* (PHZ 554) with SO obscuring parts of the primary tissue

All *Candiacervus* and *D. dama* thin sections of femora showed a similar bone tissue structure exhibiting predominantly fast-growing FLC and, when preserved, a periphery composed of a thin band of slow-growing avascular tissue (EFS), both interrupted by growth marks (Fig. 3a–d). The tissue structure of sections PHZ 660 (size one), 4809 (size two) and 2390 (size five) was badly preserved, but growth marks could be identified. In the FLC of most thin sections, we found primarily laminar and plexiform vascular orientation with scattered longitudinal canals (Fig. 3a–d). *D. dama* section PHZ 554 exhibited a small area of reticular vascularization at the medial side near the medullary cavity (Fig. 3e). The growth marks in the FLC were often surrounded by bands of slow-growing tissue (Fig. 3a, c, d). None of the cross-sections exhibited extensive amounts of Haversian bone, while all sections displayed secondary endosteal lamellar bone. A high density of secondary osteons was present in the zone of the linea aspera of all thin sections (Fig. 3f). In seven sections (2255, 2280, 2294, 3701, 4805, 4809 and *D. dama* DR001) secondary osteons substituted large parts of the primary tissue at the posterior side. Section 2015 (size five) did not retain the linea aspera, nor the posterior or medial side. Sections 2280 (size one), PHZ 430 (size one), 3701 (size three) and DR001 (*D. dama*) showed large amounts of secondary osteons scattered along the cortex, obscuring the growth record. In 2280, 3701 and DR001 even parts of the sub-periosteal area across the cortex were invaded by secondary osteons (Fig. 3c).

All-but-one section (2390, size five) had preserved an EFS. However, the majority of the cross-sections exhibited an EFS which was only visible in some regions of the cortex, due to missing parts of the periphery (2255, PHZ 660, 2015, 2390), bad tissue preservation in the outer cortex (2255, 2280, 2294, PHZ 430, PHZ 660, 4809, 2390) and/or remodelling (2255, 2280, 2294, PHZ 430, 3701, 4805, 4809, and *D. dama* DR001). In the poorly preserved sections PHZ 660 and 4809 the EFS was not easily identifiable. In PHZ 554 (*D. dama*), avascular tissue was not yet deposited at the sub-periosteal area of the posterior side, while a thin layer of avascular tissue was already present at the most external part of the lateral, anterior and medial side, indicating different bone growth rates across the cortex.

**Table 1:** Femora specimens and thin sections of Cretan deer (*Candiacervus*) and fallow deer (*Dama dama*) used in this study with age parameters

Taxon	Specimen number	Thin section number	Locality	Number of LAGs at the anterior part of the cortex within the FLC^a^	Estimated age of skeletal maturity in years	Remarks
*Candiacervus ropalophorus* *(*size one)	RGM 439541	2255	Gerani 4 cave, Crete (Greece)	5	5	
“	RGM 439545	2280	“	4	4	
“	RGM 439581	2294	“	4	4	
“	PIMUZ A/V 5195	PHZ 430	“	6	6	Specimen used in Kolb et al. (2015)
“	PIMUZ A/V 5202	PHZ 660	“	4	4*	Lateral side is not preserved. Specimen used in Kolb et al. (2015)
*Candiacervus* sp. II (size two)	RGM Li 4805	4805	Liko cave, Crete (Greece)	6	6	
“	RGM Li 4809	4809	“	5	5	
*Candiacervus cretensis* (size three)	RGM Li 3701	3701	“	6	6*	The 6th LAG anteriorly obscured by secondary osteons, but exposed medially and laterally
*Candiacervus dorothensis* (size five)	AMPG BC 2015	2015	Bate cave, Crete (Greece)	6	9	Bone fragment containing solely anterior and lateral side. The 7th to 9th LAGs only laterally preserved, due to limited cortex preservation anteriorly
“	AMPG Li 2390	2390	Liko cave, Crete (Greece)	7	7*	EFS not visible
*Dama dama*	AMPG TC DR001	DR001	Rhodos (Greece)	3	3	Wild specimen. The 1st LAG anteriorly obscured by secondary osteons, but exposed medially and laterally
“	PIMUZ A/V 5248	PHZ 554	Wildnispark Zürich (Switzerland)	2	2	Captive specimen used in Kolb et al. (2015)

^a^ The LAG forming the border between the fibrolamellar complex (FLC) and the external fundamental system (EFS) is also included

* Estimated minimal age of skeletal maturity due to an absent or questionable EFS

All specimens have fused epiphyses. Specimens and thin sections previously used in Kolb et al. (2015) are underlined. The number of lines of arrested growth (LAGs) represents total number of cyclical growth lines within the fibrolamellar complex in the anterior region

In 2294 (size one) and 3701 (size three) the boundary between the FLC and avascular tissue in the sub-periosteal zone seems to differ depending on the region of the cortex. In 2294, avascular tissue was observed beyond the fourth LAG anteriorly and laterally, whereas in the posteromedial region, non-vascular tissue was already deposited beyond the third LAG. Similarly, in 3701, no vascular canals were recognized anteriorly and laterally after the sixth LAG, while medially avascular tissue was already present beyond the fourth LAG. As solely non-vascular tissue was deposited beyond the fourth LAG in 2294 and the sixth LAG in 3701 throughout the entire cortex, the EFSs begin from those growth marks. In specimens exhibiting an absent or questionable EFS (PHZ 660, 4809, Li 2390), we suggest caution and recommend referring to the minimal age of skeletal maturity rather than the exact age to avoid possible misinterpretation (Table 1).

Not all LAGs could be reconstructed at the anterior side of the sections due to limited cortex preservation in 2015 or secondary remodelling in 3701 and DR001. Here, we identified LAGs at other regions of the cortex (Table 1). We counted the seventh to ninth LAGs in thin section 2015 laterally (Fig. 3d), because anteriorly, a large part of the cortex preserving these lines had broken off. Furthermore, the cortex of five *Candiacervus* sections of sizes one (2255, PHZ 430), three (3701) and five (2015, 2390) showed medullary expansion towards the lateral side, partially erasing the first LAG. In PHZ 430 also the second LAG, which is relatively close to the first one, is partly eroded laterally. Comparing the growth record of the different cross-sections within each taxon shows that there was probably no complete elimination of LAGs due to expansion of the medullary cavity. In the two *D. dama* sections no erosion of LAGs was observed.

The bone cortices of the sampled dwarf and medium size *Candiacervus* (sizes one, two and three) show similarity in the ages of attainment of skeletal maturity (Table 1). These are four to six years for the smallest size (size one n = 5), five to six years for the one-but-smallest size (size two; n = 2) and a minimum of six years for the medium size (size three; n = 1). The one-but-largest *Candiacervus* specimens seems to indicate skeletal maturity at seven to nine years of age (size five; n = 2). This implies that all sampled specimens of *Candiacervus*, irrespective of size, reached skeletal maturity at a later age than our *D. dama* specimens, which were skeletally mature at two to three years (n = 2). The largest *Candiacervus* taxon (size five) included here seems to have had the slowest maturation. However, a cautionary note is needed here, as these observations are based on a small number of specimens.

## Discussion

All studied femora of the Pleistocene deer of Crete (*Candiacervus*) and recent fallow deer (*D. dama*) exhibited a bone tissue microstructure similar to that observed in other deer, including mainland red deer, *Cervus elaphus* (Calderón et al., 2019; Jordana et al., 2016) and mainland and insular forms of sika deer, *Cervus nippon* (Hayashi et al., 2023). Additionally, the structure of the primary bone tissue observed here, consisting mainly of laminar and plexiform FLC, is in accordance with earlier results for size one and two *Candiacervus* (Kolb et al., 2015). On the other hand, other fossil Pleistocene insular deer (Ryukyu dwarf deer, *Cervus astylodon* and the Ryukyu muntjac, Muntiacini gen. et sp. indet.) exhibit mainly slow-growing parallel-fibred bone (Hayashi et al., 2023), whereas the extinct Plio-Pleistocene Balearic Islands cave goat, *Myotragus balearicus*, shows primarily slow-growing lamellar bone (Köhler & Moyà-Solà, 2009). The predominant presence of slow-growing tissues implies adaptation to a slow life history (Hayashi et al., 2023; Köhler & Moyà-Solà, 2009). The tissue type of the studied *Candiacervus* specimens thus does not seem to indicate a slow life history relative to mainland deer (*D. dama*, *C. elaphus* and *C. nippon*).

In addition to lower rates of bone deposition, delayed maturation is also an indicator of a slow life history strategy (Hayashi et al., 2023; Köhler & Moyà-Solà, 2009; Köhler et al., 2021; Long et al., 2019; Palkovacs, 2003). Our results indicate that the one-but-largest *Candiacervus* attained skeletal maturity at a later age than the similarly tall extant red deer, the much larger extant moose, *Alces alces*, as well as the extinct giant deer, *Megaloceros giganteus* (Table 2). Our results on the two smallest *Candiacervus* indicate a somewhat earlier attainment of skeletal maturity (4–6 years) compared to earlier research (5–7 years: Kolb et al., 2015), but still displayed a later timing of skeletal maturity than both the similarly-sized mainland roe deer, *Capreolus capreolus* and the much larger moose (Table 2). Furthermore, both dwarf *Candiacervus* exhibited a similar timing as the much larger extinct giant deer and extant red deer (Table 2). The variation in timing of skeletal maturity per size seen in *Candiacervus* may theoretically be due to sexual dimorphism as found in mainland deer (as shown in Calderón et al., 2019; Flinn et al., 2013). Our results, in comparison with earlier research, seem to imply that the evolution of gigantism and dwarfism in *Candiacervus* correlates with a slower and prolonged growth relative to its closest living relative (*D. dama*) in both directions of evolutionary size shifts. The medium size *Candiacervus* also shows a delayed attainment of skeletal maturity relative to the similarly-sized and putative ancestral fallow deer (Table 2). This may indicate that the *Candiacervus* species, which retained the ancestral size evolved slower and prolonged growth as well. Our results are in agreement with those found for long bones of other island ruminants including the Ryukyu dwarf deer (Table 2), the Ryukyu muntjac (Table 2) and Balearic Islands cave goat (over 12 years: Köhler & Moyà-Solà, 2009), which all indicate a similar or even later timing of skeletal maturity than observed in our *Candiacervus* sample. The delayed skeletal maturity in *Candiacervus* relative to similar-sized mainland deer supports our hypothesis of a slower life history relative to mainland deer.

**Table 2:** Timing of skeletal maturity of island and mainland deer. The average adult body masses are taken from Kolb et al. (2015), Besiou et al. (2022) and Hayashi et al. (2023)

Taxon	Island/mainland	Body mass	Timing of skeletal maturity	References
Muntiacini gen. et sp. indet	Island	16.5 kg	5–11 years	Hayashi et al. (2023)
*Capreolus capreolus*	Mainland	21.7 kg	3 years	Kolb et al. (2015)
*Cervus astylodon*	Island	25.4 kg	9–16 years	Hayashi et al. (2023)
*Candiacervus ropalophorus* (size one)	Island	27.8 kg	4–6 years	This study
*Candiacervus* sp. II (size two)	Island	41.5 kg	5–6 years	This study
*Dama dama*	Mainland	70.0 kg	2–3 years	This study
*Candiacervus cretensis* (size three)	Island	74.7 kg	6 years	This study
*Candiacervus dorothensis* (size five)	Island	170.1 kg	7–9 years	This study
*Cervus elaphus*	Mainland	200.0 kg	4–6 years	Kolb et al. (2015); Calderón et al. (2019)
*Alces alces*	Mainland	386.0 kg	3 years	Kolb et al. (2015)
*Megaloceros giganteus*	Mainland	630.0 kg	5–6 years	Kolb et al. (2015)

Overall, somewhat earlier timing of skeletal maturity in *Candiacervus* compared to other island ruminants, seems to indicate that *Candiacervus* did not undergo a strong shift towards a slow life history. In addition, the similarity in bone tissue structure between *Candiacervus* and mainland deer, contrasting with the tissue types typically associated with slow growth in other insular ruminants, supports the presumable limited shift towards a slow life history in dwarf, medium, as well as giant sizes of *Candiacervus*. This limited shift may be due to two factors. Firstly, *Candiacervus* underwent a much shorter time of evolution in isolation (< 0.25 Ma: Besiou et al., 2022). The insular ruminants with a more pronounced shift towards a slow life history lived much longer in isolation: over 1.5 million years for the Ryukyu dwarf deer and Ryukyu muntjac (Hayashi et al., 2023) and about 5.2 million years for *Myotragus balearicus* (Köhler & Moyà-Solà, 2009). Secondly, high intraspecific competition within each *Candiacervus* taxon may have hampered ecological release and prevented the acquisition of traits that are more typical for anagenetic insular lineages. In order to test for the effect of these two factors, further study is needed of insular species with differences in evolutionary time as well as intraspecific competition within insular deer taxa.

## Conclusions

Our study indicates a moderate shift towards a slow life history in an insular deer lineage that underwent cladogenesis (species radiation): the Pleistocene deer of Crete (*Candiacervus).* Dwarf, medium as well as giant sizes of *Candiacervus* seem to have evolved delayed skeletal maturity, as based on the number of growth lines (LAGs) in bone cortex, which suggests a combination of slow and prolonged growth to reach adulthood, probably as a response to limited resources, and reduced levels of predation and competition. This is the first report on the attainment of a slower life history, as inferred from delayed skeletal maturity, in an insular deer species radiation including species of ancestral size, dwarfed and giant species. The moderate extent to which *Candiacervus* evolved a slower maturation in comparison with anagenetic insular ruminant lineages may be due to a limited time in isolation, high intraspecific competition within a *Candiacervus* taxon or a combination of these factors.

## Data Availability

The examined specimens and thin sections are stored in the collections of the Athens Museum of Palaeontology and Geology (National and Kapodistrian University of Athens, Greece), Naturalis Biodiversity Centre (Leiden, the Netherlands) and Paläontologisches Institut und Museum (Universität Zürich, Switzerland).

## Abbreviations

AMPG: Athens Museum of Palaeontology and Geology, National and Kapodistrian University of Athens, Greece
RGM: Naturalis Biodiversity Centre, Leiden, the Netherlands (formerly Rijksmuseum voor Geologie en Mineralogie)
PIMUZ: Paläontologisches Institut und Museum, Universität Zürich, Switzerland
